# *CDKN2A* c.146T>C (I49T) in Mexican-ancestry individuals: Findings from melanoma and population cohorts

**DOI:** 10.1016/j.jdin.2026.09.001

**Published:** 2026-09-15

**Authors:** Brooke Bartley, Ariel B. Brown, Amr Salem, Isabella Ribaudo, Sophia N. Wix, Marelize Snyman, Jacqueline A. Mersch, Amber P. Gemmell, Rebecca Vasquez, Kelly C. Nelson, Joshua Arbesman, Jennifer G. Gill

**Affiliations:** a Department of Dermatology, UT Southwestern Medical Center, Dallas, Texas; b Department of Dermatology, Medical Specialty Institute, Cleveland Clinic, Cleveland, Ohio; c Cancer Genetics Program, Simmons Comprehensive Cancer Center, UT Southwestern Medical Center, Dallas, Texas; d Department of Dermatology, MD Anderson Medical Center, Houston, Texas

**Keywords:** cancer genetics, CDKN2A, health care disparities, melanoma, pancreatic cancer

*To the Editor:* Melanoma occurs less frequently in Hispanic individuals than in non-Hispanic White populations; however, Hispanic patients often present with more advanced disease and experience worse outcomes. The determinants of melanoma susceptibility in Hispanic populations remain poorly defined, suggesting potential contributions from underrecognized inherited risk variants.

*CDKN2A*, encoding tumor suppressors p14 and p16, is the most well-characterized hereditary melanoma susceptibility gene and is associated with increased risk of melanoma and pancreatic cancer. However, most studies have focused on European ancestry populations, and its contribution in Hispanic individuals remains unclear.

Here, we report the *CDKN2A* c.146T>C (p.Ile49Thr, I49T) variant among Hispanic melanoma patients at our institution and evaluate population-level datasets to define its ancestry distribution and cancer associations. Detailed methods are available in Supplementary Material, available via Mendeley at https://data.mendeley.com/datasets/4f5vvd7sdn/2.

We developed a retrospective patient registry of 211 Hispanic melanoma patients treated at UT Southwestern Medical Center and Parkland Memorial Hospital between 2006 and 2022.^1^ Among 101 Hispanic patients with nonacral cutaneous melanoma, 6 underwent germline *CDKN2A* testing, of whom 3 (50%) carried I49T. Although this clinically selected subset cannot provide an unbiased estimate of variant prevalence, the unexpectedly high frequency of I49T prompted further investigation in population-level datasets. All 3 carriers were Hispanic women diagnosed in their 30s–40s with a single melanoma and no prior cutaneous malignancies (Table I). Each had an additional malignancy (1 pancreatic and 2 breast cancers), and only 1 met conventional criteria for *CDKN2A* testing based on personal or family history.

**Table I:** Characteristics of 3 Hispanic patients with melanoma and *CDKN2A* c.146T>C (I49T) variant

Patient	Age	Sex	Melanoma subtype (site)	Prior melanoma/Skin cancer history	Dysplastic nevi	Personal cancer history	Family cancer history
1	40s	F	Lentigo maligna (neck)	None	Present	Pancreatic cancer	Mother: colon cancer
2	30s	F	Melanoma in situ (shoulder)	None	None	Breast cancer	None
3	30s	F	Superficial spreading melanoma (neck)	None	None	Breast cancer	Family history of cancer, type unknown

Population-level analysis using the PAGE consortium demonstrated a highly ancestry-specific distribution, with I49T allele frequencies of 0.435% in individuals of Mexican ancestry and 0.080% in individuals of Native American ancestry, corresponding to estimated carrier frequencies of approximately 0.87% and 0.16%, respectively. The variant was not detected in other Latin American groups (Supplementary Table I, available via Mendeley at https://data.mendeley.com/datasets/4f5vvd7sdn/2).^2^

In the All of Us Research Program, 342 of 57,845 participants of Mexican ancestry (∼0.6%) carried I49T.^3^ Carriers had a significantly higher prevalence of cancer overall than noncarriers (7.6% vs 3.7%; OR 2.10, *P* < .001), with increased prevalence also observed across several individual malignancies, including oropharyngeal, genital, lung, and gastric cancer (Table II). Melanoma prevalence was higher among carriers (odds ratio: 5.46) but did not reach statistical significance because of low event numbers.

**Table II:** Cancer prevalence in All of Us Research Program database among *CDKN2A* c.146T>C (I49T) carriers and noncarriers reporting Mexican ancestry

Cancer type	Noncarrier prevalence [*n*/*N* (%)]	Carrier prevalence [*n*/*N* (%)]	Odds ratio	Adjusted *P*-value
Oropharyngeal	54/57,503 (0.1%)	4/342 (1.2%)	11.61	7.6e-05
Genital	93/57,503 (0.2%)	4/342 (1.2%)	7.15	.002
Lung/Bronchus	88/57,503 (0.2%)	3/342 (0.9%)	5.73	.029
Stomach	44/57,503 (0.1%)	2/342 (0.6%)	7.45	.037
Breast	407/57,503 (0.7%)	6/342 (1.8%)	2.78	.081
Cancer overall	2137/57,503 (3.7%)	26/342 (7.6%)	2.10	4.02e-04

Collectively, our institutional and population-level data demonstrate I49T as an ancestry-enriched variant in individuals of Mexican ancestry, with a carrier frequency of approximately 0.6% to 0.9% across 2 large datasets. A recent study of Mexican families and hereditary cancer patients independently raised the possibility of a Mexican founder effect and reported a broader cancer phenotype among I49T carriers.^4^ Although I49T has conflicting classifications in ClinVar, several clinical laboratories now classify it as likely pathogenic based on familial studies and in vitro functional evidence.^5^ However, its penetrance and variant-specific cancer risks remain incompletely defined.

While pathogenic *CDKN2A* variants have classically been associated with melanoma and pancreatic cancer, I49T may be associated with a broader cancer phenotype. Notably, the recent Mexican cohort reported breast cancer as the most frequent malignancy among carriers, while our population-based analysis demonstrated significantly increased cancer prevalence overall, including several individual cancer types although this should be considered exploratory given the small number of carrier events.^4^ Additional studies are needed to define variant-specific cancer risks and implications for preventative screening.

The relatively high population frequency of I49T, together with emerging evidence of cancer association, suggests that some carriers of Mexican ancestry may not be identified using conventional phenotype- and family history-based *CDKN2A* testing criteria. Further studies are needed to define melanoma penetrance, broader cancer risks, and whether ancestry should ultimately inform genetic testing strategies.

### Declaration of generative AI and AI-assisted technologies in the writing process

Artificial intelligence tools were used solely for language editing.

## Conflicts of interest

None disclosed.
